# Is the Oxidative Stress in Obstructive Sleep Apnea Associated with Cardiovascular Complications?—Systematic Review

**DOI:** 10.3390/jcm9113734

**Published:** 2020-11-20

**Authors:** Piotr Fiedorczuk, Adam Stróżyński, Ewa Olszewska

**Affiliations:** 1 Doctoral School of the Medical University of Bialystok, 15-328 Białystok, Poland; piotr.fiedorczuk@umb.edu.pl; 2 Medical University of Bialystok, 15-328 Białystok, Poland; ad.strozynski@gmail.com; 3 Department of Otolaryngology Medical University of Bialystok, 15-328 Białystok, Poland

**Keywords:** Sleep apnea, oxidative stress, cardiovascular disease

## Abstract

Obstructive sleep apnea (OSA) is a prevalent, underdiagnosed disease and is considered an independent risk factor for cardiovascular disease. The exact mechanism of cardiovascular complications (CVC) development as a complication of OSA is not entirely understood. Oxidative stress is suspected to be the essential factor in initiating various comorbidities in OSA. Biomarkers of nonenzymatic lipid and protein peroxidation, DNA repair and antioxidant capabilities measured in serum, plasma and urine are frequently used to assess the presence of oxidative stress. We conducted a systematic review and quality assessment of available observational analytic studies to determine whether there is an association between oxidative stress and OSA in patients with prevalent CV disease compared to (a) patients with prevalent CV disease but no OSA, (b) patients with prevalent CV disease and less severe OSA and (c) patients with OSA and no overt CV disease. This systematic review demonstrated that, while oxidative stress is associated with OSA, there was no clear difference in the severity of oxidative stress between OSA patients with or without cardiovascular complications.

## 1. Introduction

Obstructive sleep apnea (OSA) is a nocturnal disorder characterized by recurrent episodes of upper airway obstruction during sleep resulting in oxygen desaturation and sleep fragmentation. Various treatment and diagnosis methods of OSA are available [1,2,3,4,5]. It is an increasingly prevalent condition that has a great impact on public health. Epidemiologic data shows it as a disorder with high prevalence estimated to be 9% to 38% in the general adult population, from 13% to 33% in men and from 6% to 19% in women, with the disease being more frequent in men and older age groups [6]. Patients with untreated OSA are at increased risk of hypertension, cardiovascular disease, heart failure, obesity, metabolic dysregulation, diabetes mellitus, daytime sleepiness, depression, accidents, stroke and dementia [7,8,9,10].

### 1.1. Oxidative Stress in Obstructive Sleep Apnea

The pathogenesis of complications in OSA is multifactorial and not fully established. It involves a diverse range of mechanisms, including selective activation of inflammatory molecular pathways, endothelial dysfunction, metabolic dysregulation and oxidative stress [11].

In OSA, the recurrent obstruction of the upper airway during sleep results in cycles of significant hypoxia exaggerated negative intrathoracic pressure and arousals. Repetitive hypoxia and re-oxygenation induce excess creation of reactive oxygen species (ROS). This causes oxidative stress, which is an imbalance between the production of oxygen free radicals and the antioxidant capacity of the organism. Higher generation of ROS can occur in normal aging or in acute or chronic pathophysiological conditions. The excess of ROS causes oxidative damage to deoxyribonucleic acid (DNA), proteins and lipids [12].

Biomarkers of nonenzymatic lipid and protein peroxidation, DNA repair and antioxidant capacity measured in serum, plasma and urine are commonly used to assess the presence of oxidative stress. [13]. Oxidative stress is responsible for the alteration of substantial amounts of measurable compounds, a summary of which is provided in Table 1. One must keep in mind that this list of oxidative stress biomarkers is surely incomplete and ever-growing. The list was selected to include only established and widely tested biomarkers of oxidative stress in OSA.

### 1.2. Cardiovascular Complications in Obstructive Sleep Apnea

Studies show that OSA increases the risk of cardiovascular complications (CVC), such as the development or progression of arterial hypertension, ischemic heart disease, heart failure and stroke [40]. A study published by Peppard et al. [41], which included 709 participants who completed the four-year follow-up study and 184 who also completed the eight-year follow-up study, reported that patients suffering from mild OSA (AHI < 15) and severe OSA (AHI ≥ 15) had respectively about two and three times, higher odds of having hypertension at follow-up of those free of disease. In a 2005 study published in The Lancet, Marin et al. presented a large, prospective controlled study with a mean of 10.1 years of follow-up. The study group consisted of 264 healthy men, 377 simple snorers, 403 with untreated mild-moderate obstructive sleep apnea–hypopnea, 235 with untreated severe OSA and 372 patients treated with CPAP. The results of the study suggest that for men with untreated severe OSA, the odds ratio of fatal and non-fatal cardiovascular events is 2.87 and 3.17, respectively [42]. Gottlieb et al. in 2010 published a prospective study with an 8.7-year median follow-up of 1927 men and 2495 women ≥ 40 years free of heart failure and coronary heart disease. After adjusting for multiple risk factors, OSA was a significant predictor of incident coronary heart disease among men (men aged 40–70, who had AHI ≥ 30 were 68% more likely to establish coronary heart disease than those with AHI < 5) [43].

Oxidative stress and chronic inflammatory state are thought to be the main characteristic of pathophysiological changes in OSA contributing in consequence to the neural, cardiovascular and metabolic alterations. Oxidative stress in sleep apnea patients may be the main cause of endothelial dysfunction [44]. Endothelial dysfunction is often considered as one of the earliest detectable and possibly reversible abnormalities during the development of atherosclerosis. Studies indicate an association between the presence of both coronary and systemic endothelial dysfunction and an increased risk for future cardiovascular morbidity and mortality in patients with OSA [45,46,47]. Moreover, the development of cardiovascular morbidity and mortality may also occur secondary to other pathologies caused by OSA, such as hypertension [48].

### 1.3. Objective of This Study

It is still unknown if oxidative stress is responsible for cardiovascular complications in untreated OSA patients. Patients with hypertension, ischemic heart disease and other CVCs may or may not have higher levels and concentrations of biomarkers of oxidative stress compared to non-OSA controls or CVC-free OSA patients.

This systematic review aims to investigate whether patients suffering from OSA demonstrate oxidative stress in association with cardiovascular complications

## 2. Methods

The criteria of preferred reporting items for systematic reviews and meta-analysis (PRISMA) Checklist [49] were followed in conducting and reporting of this systematic review. Our PICO (Population, Indicator, Control, Outcome) question is shown in Table 2:

We searched Pubmed and Scopus Library for non-review articles concerning sleep apnea, oxidative stress and antioxidants. The search was performed using the words “sleep apnea”, “disordered breathing”, “oxidative” and “antioxidants” in different combinations.

We searched Pubmed database using a following string: (((sleep apnea) AND (oxidative)) OR ((disordered breathing) AND (oxidative))) OR ((sleep apnea) AND (antioxidants)) OR ((disordered breathing) AND (antioxidants).

To obtain literature from the Scopus library, we used the following string:

TITLE-ABS-KEY ((((sleep AND apnea) AND (oxidative)) OR ((disordered AND breathing) AND (oxidative))) OR ((sleep AND apnea) AND (antioxidants)) OR ((disordered AND breathing) AND (antioxidants))) AND (LIMIT-TO (DOCTYPE, &quot;ar&quot;)) AND (LIMIT-TO (SUBJAREA, &quot;MEDI&quot;)).

Search results were exported to the Mendeley reference manager for the initial title and abstract screening of the records. Duplicate articles were removed by the “remove duplicates” function of Mendeley [50]. The literature search was performed between 15th June 2020 and 21st June 2020. To obtain articles not received from databases, bibliographies of published articles were manually reviewed to identify additional studies. Two authors independently performed the literature search, evaluated articles for inclusion and discrepancies, if any, were resolved with discussion.

**Figure 1:**
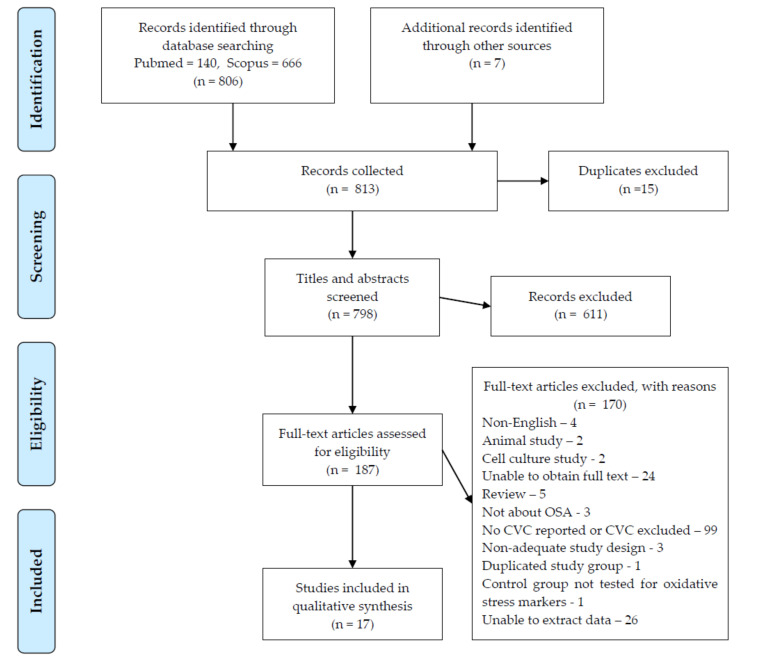
Prisma flow diagram OSA, Obstructive Sleep Apnea; CVC, cardiovascular complications.

During the initial screening of titles and abstracts, the studies retrieved had to meet the following criteria for inclusion in full-text eligibility assessment: (1) non-review articles (2) papers concerning adult human subjects with OSA (3) measurement of chosen oxidative stress biomarkers (Table 1) After the initial screening, full-text manuscripts were retrieved and independently assessed by two investigators. The process for selecting the studies is provided in the flow chart (Figure 1). A quality score evaluation for studies was done according to the Newcastle–Ottawa scale (NOS) for case–control/observational studies [51].

## 3. Results

Our search overall included 806 articles. After applying our criteria and excluding papers that did not meet them, we selected and analyzed 17 publications (Figure 1). Every reviewed article involved a chosen oxidative stress biomarker.

Articles satisfying inclusion criteria for this systematic review, the following information was extracted: first author names, year of publication, oxidative stress biomarkers tested, AHI scoring criteria, OSA case definition, CVC reported, population and Newcastle–Ottawa scale. Quality assessment of studies to a maximum of nine points included three components: selection, comparability and exposure.

To clearly analyze our results and properly connect them with the aim of the study, we grouped articles into 3 categories:

1. OSA vs. non-OSA control;
2. severe OSA vs. non-severe OSA;
3. OSA with CVC vs. OSA without CVC.

According to this division, we also extracted: number of OSA and control subjects, percentage of males, means and SD of age, BMI and AHI, biomarkers related to oxidative stress measured and data regarding patients with cardiovascular comorbidities. The foregoing biometric characteristics were used to compare groups of patients presented in the articles. Data regarding the basics of chosen studies as well as the Newcastle–Ottawa scale (NOS) score is presented in Table 3.

We assessed the quality of the studies based on the selection of subjects, comparability of cases and controls and the subject’s exposure (detailed scoring in Table 3). The majority (10/17) of studies scored 7 points out of a maximum of 9; a suggested cut off value for “good quality”, with the lowest NOS score of 5 (2/17 studies). With the mean score of all selected studies of 6.8 out of 9, the studies present fairly good overall quality. Nevertheless, while NOS is a useful and convenient tool commonly used in systematic reviews, one must take into consideration its limitations [52]. While two studies sharing the same score in NOS may have an entirely different risk of bias and implication for future research, we analyze the appropriate methodological aspects individually under each category (Table 3).

### 3.1. OSA Group vs. Non-OSA Control Group

In this category, we grouped papers describing patients with OSA compared to non-OSA control patients with the same or similar cardiovascular complications rate in both groups. Studies from this group reported the extent of oxidative stress, measured by various plasma and urinary oxidative stress biomarkers in patients with OSA compared to non-OSA controls. Most of the groups were similar in biometric aspects.

Anunciato et al. compared hypertensive patients with and without OSA and reported no difference in the level of NO as well as no differences in biometric characteristics between the two groups [53].

Cherneva et al. and De Lima et al. reported a correlation between oxidative stress biomarkers and OSA and no significant difference in HT percentage between groups. Groups were also similar, considering other factors [54,55].

Del Ben et al. presented a difference in 8-isoprostane levels between the severe OSA group and the control, mild/moderate groups (*p* < 0.001) with no disparity in NOx levels. There was a difference in BMI between the severe OSA group and mild/moderate OSA group (*p* = 0.03), but also between control and severe OSA groups (*p* = 0.001) [56].

Monneret et al., who studied metabolic syndrome patients with and without OSA similar in biometric characteristics, reported no statistical differences in biomarker levels between groups other than homocysteine (*p* = 0.026) [57].

Piérola et al. showed a significant difference in 8-isoprostane levels between OSA and non-OSA control groups (*p* = 0.001), although he considered groups with major differences in biometric characteristics and HT prevalence (*p* < 0.001) [58].

Wang et al. divided patients into 4 groups: elderly with OSA, elderly without OSA, non-elderly with OSA, non-elderly without OSA. They presented differences in Hcy, MDA and GSH levels among groups. Groups were similar, considering other factors [59].

Ortaç Ersoy et al. divided patients into four groups: OSA, CAD, OSA + CAD and a healthy control group; therefore, this article could be matched with two categories. He showed no difference between CAD and OSA + CAD groups, but also no statistical difference in biomarker levels [60]. Patients participating in the study were also grouped as having one-, two- or three-vessel CAD. Most of the patients in the CAD group had one or two vessels afflicted, whereas participants in the OSA + CAD group suffered mostly from two or three-vessel disease [55].

In summary, the majority of researchers confirmed a relationship between increased oxidative stress biomarker levels and OSA regardless of existing CVC. The results of this comparison further cement OSA—and not the presence of cardiovascular comorbidities—as the cause of oxidative stress. This conclusion has a crucial role in interpreting the results of the other two comparisons in this study (Table 4).

### 3.2. Severe OSA vs. Non-Severe OSA

This category contains articles comparing patients with different disease severity. Studies grouped in this category depicted more differences in biometric characteristics than in the previous category. It was not obligatory to include a non-OSA control group, as comparisons would be made between at least two OSA groups, preferably with a similar prevalence of CVC.

Chen et al. described patients with a history of ischemic stroke and divided them into two groups with AHI > 30 and AHI < 30 and presented no statistical difference in biomarker levels, but with an age difference (*p* = 0.002) and HT prevalence (*p* = 0.0021) [61].

Gille et al. reported significant differences in 8-OHdG levels between both moderate and severe OSA groups compared to the group with AHI < 15. The levels of MMP-7 in the severe OSA group were significantly higher compared to other groups. There were also no differences in biometric characteristics among groups except for the difference in age between the no OSA group and the moderate OSA group [62].

Yadav et al. presented two groups with high and low AHI and no difference between groups considering other biometric factors; however, patients were divided around median AHI, not according to OSA staging. He showed relevant differences in PON1 levels (*p* < 0.0001) with no disparity in HT prevalence [63].

Yamauchi et al. compared two groups with non-severe and severe OSA with no significant differences except for BMI (*p* < 0.001) and showed a difference in 8-OHdG levels (*p* = 0.023), but with no disparity in HT percentage [64].

Yardim-Akaydin et al. divided patients into 4 groups: healthy controls, mild apnea, moderate apnea and severe apnea. Moderate and severe apnea groups were significantly older than the mild apnea and control groups (*p* < 0.001). BMI of the severe apnea group was higher than in moderate, mild apnea and control groups (*p* < 0.05), but there were no statistical differences in HT prevalence among groups except for the disparity in HT prevalence between severe apnea and control group. They noted a significant difference in 8-OHdG levels between severe and mild OSA groups (*p* = 0.029), but also between both moderate and mild apnea groups compared to the control group (*p* < 0.05). The only difference in the MDA level was between the control group compared to severe, moderate and mild apnea groups (*p* < 0.05). After adjusting the biomarker levels for BMI, hypertension, diabetes mellitus and hyperlipidemia, the MDA levels in the control group were lower than all other OSA groups; mild, moderate and severe (control 2.25 ± 0.23 vs. mild 3.12 ± 0.08 vs. moderate 3.17 ± 0.15 vs. severe 3.19 ± 0.15 µM). The adjusted values of 8-OHDG shown no difference between OSA groups. However, they were higher in every OSA group compared to controls (control 25.55 ± 3.76 vs. mild 31.72 ± 4.01 vs. moderate 32.06 ± 5.14 vs. severe 32.48 ± 4.26 nM/creatinine mM) [65].

All but one of the selected studies from this category shows a positive correlation between oxidative stress biomarker levels and the severity of OSA. This effect is also present in groups with similar CVC prevalence. Nevertheless, these findings are limited by disparities in biometric characteristics among groups (Table 5).

### 3.3. OSA with CVC vs. OSA without CVC

In this category, we included articles that reported the levels of oxidative stress biomarkers in OSA patients with cardiovascular complication or comorbidity and OSA patients free of CVC.

Feres et al. divided patients into 3 groups: OSA with comorbidities, OSA without comorbidities, healthy controls. The inclusion criteria for the group with comorbidities were hypertension and dyslipidemia. There were no significant differences between the two OSA groups. They also showed no significant difference in oxLDL levels between the two groups. Instead, they noted differences in age between OSA with and without comorbidities groups compared to the control group (*p* < 0.001) as well as differences in BMI between OSA with comorbidities group and control group (*p* = 0.0006) [66].

İn et al. presented three groups: OSA with CVD risk factors, OSA without CVD risk factors and healthy control group. Patients were categorized into OSA with CVD risk factors if they had at least one of the following risk factors: obesity, hypercholesterolemia, diabetes, hypertension and smoking, however according to mean BMI and SD at least some of the patients from OSA without CVD risk factors group could be classified as obese. Groups were not matched in the terms of age (*p* < 0.01) and BMI (*p* < 0.0001) with the group with the aforementioned risk factors being older and with higher BMI. Oxidative stress biomarkers results showed relevant differences in ADMA levels (*p* < 0.01) between the OSA groups and control groups, but the difference between OSA with CVD risk factors and OSA without CVD risk factors group was not significant [67].

Lavie et al. divided patients into three groups: OSA + CVD, OSA − CVD and a healthy control group. She considered IHD, MI and HT as CVD. There were differences in biometric characteristics between the two OSA groups. The OSA + CVD included older patients (*p* < 0.0001) and had higher BMI (*p* < 0.05) than OSA − CVD patients although with similar OSA severity, as there was no difference in AHI. They also noted differences in age between the OSA + CVD group and the control group (*p* < 0.0001) as well as differences in BMI between both OSA groups compared to the control group (*p* < 0.001). Both OSA groups had higher levels or concentrations of oxidative stress biomarkers in comparison to a healthy control group, but the difference between OSA + CVD and OSA − CVD was not significant. It’s important to note the relatively high mean AHI of the control group (8.2 ± 2.6) which implies mild OSA in at least a few of the control subjects. [68].

Murri et al. presented a group of OSA patients, which he later divided into two groups including hypertensive patients with OSA and normotensive patients with OSA. Biometric characteristics were provided for the whole study population, but not for divided groups. There was a difference between groups considering CAT and TAC levels (*p* < 0.05), but none in GPx and SOD levels [69].

Ortaç Ersoy et al. that was matched in two categories presented no significant differences between groups except for AHI, which was higher in the OSA group than in the OSA + CAD group (*p* = 0.005) and no statistical difference in oxidative stress biomarker levels [60]. The aforementioned disparity in AHI allows classifying the OSA group as severe OSA and OSA + CAD as moderate.

In summary, the researchers presented no relation between oxidative stress biomarker levels and CVC prevalence in OSA patients or the relationship did not correspond with all biomarkers measured. Nonetheless, the vast majority of studies still included groups with disparities in biometric characteristics among them (Table 6).

## 4. Discussion

The state of oxidative stress can occur not only in OSA patients but also in various pathological conditions, e.g., cancer, neurological and respiratory disease or even depression [70]. Whatever the cause, it is associated with a harmful effect on cell membranes, DNA structure and proteins, causing conformational modifications and damage. In OSA, it is theorized that through ROS overproduction and insufficient antioxidant defense, oxidative stress causes cardiovascular complications. Perhaps this relationship is not as simple as it was previously suggested. This is the first systematic review of the association between oxidative stress and cardiovascular complications in OSA.

Available studies provide compelling evidence that OSA can be characterized as an oxidative stress disease, whether by increased production of superoxide anion in OSA patients’ white blood cells (neutrophils, stimulated and non-stimulated monocytes) [71,72] or in elevated markers of lipid peroxidation, protein carbonylation and DNA oxidation [73].

In our systematic review, when comparing OSA subjects to non-OSA controls, the majority of studies reported higher levels and concentrations of biomarkers of oxidative stress and a correlation between the levels and OSA severity, regardless of the CVC prevalence in the control groups. This solidifies the role of oxidative stress in OSA but also underlines that in non-OSA patients, either oxidative stress is not related to cardiovascular comorbidities or the sole presence of hypertension, metabolic syndrome, coronary artery disease, or ischemic heart disease is not sufficient to promote oxidative stress.

Studies comparing patients with severe and non-severe OSA gave rise to more questions than answers. In the second category, we still observe some positive relation between the severity of the disease and oxidative stress biomarkers, regardless of CVC prevalence in groups, although these results may be influenced by the disparity in groups regarding age or BMI. This may further solidify the notion that the extent of oxidative stress is not correlated with the presence of CVC or the relationship between the severity of OSA, oxidative stress and cardiovascular complications are complex.

Our last category compared OSA cases with cardiovascular complications or comorbidities and those free of CVC. This category most adequately tackles the question of whether patients with OSA have increased levels of oxidative stress biomarkers with cardiovascular complications such as arterial hypertension or ischemic heart disease. By comparing OSA groups matched by age, BMI and AHI (factors are known to increase levels of oxidative stress biomarkers), one can expect that OSA patients with comorbidities present a greater extent of oxidative stress. Provided research does not fully support this theory, as four out of five studies did not report differences in levels of oxidative stress biomarkers in both groups, even between groups with biometrical differences (age, BMI, AHI).

Analyzing the results of this systematic review, we conclude that oxidative stress may play a part in the development of cardiovascular comorbidities in OSA, but the extent of it, as measured with various plasma, serum and urinary biomarkers, does not seem to be related to the presence of cardiovascular complications including hypertension. This observation has implications on the OSA risk stratifying, therapy and prevention. Measuring a patient’s lipid peroxidation, protein peroxidation, DNA repair biomarkers, or antioxidant status may suggest the severity of OSA but may not provide insight into the cardiovascular complications. Researchers should consider the results of this review when designing future studies. Assigning the decrease in levels of oxidative stress biomarkers as a surrogate outcome for reducing cardiovascular complication risk may not fully reflect the desired result.

### Study Limitations

The topic of oxidative stress in OSA is not a novel one, and the number of studies regarding it is substantial. Despite our efforts in selecting the most attuned articles, a few limitations of this systematic review require mentioning.

The sample size of chosen studies is considerably small, with only 8 out of 17 exceeding 100 subjects. Small study samples diminish the statistical power of the research and might be the reason to question the results.

There were differences in the biometric characteristics of the groups. Del Ben et al., Pierola et al., Gille et al., Yamauchi et al., Feres et al. Lavie et al. all reported differences in age and BMI, factors are known to escalate oxidative stress and influence the biomarker levels [56,58,62,64,66,68]. As for the CVC prevalence and AHI among groups, these need to be considered according to the study category. When comparing OSA patients to non-OSA controls or patients with different severities of OSA, CVC prevalence should be similar for all groups. With the third category, at least one group of patients free of CVC or cardiovascular risk factors was required. Patients in the third category should have similar AHI, and patients in the first and second categories should be grouped according to the severity of the disease. Only one study (Yardin-Akaydin et al.) provides biomarker results adjusted for BMI and comorbidities of the studied groups. In other studies, either the adjustments were only mentioned in the text but not provided concerning various regression coefficients between the biomarker results and PSG results or not made at all.

Many authors focus on AHI as a definite predictor of disease severity. This is commonly considered true but may not be the best predictor of the state of oxidative stress or CVC risk in a patient’s body. For example, a study by Punjabi et al. [74] found that only hypopneas with a 4% or more decrease in saturation are associated with cardiovascular disease, and hypopneas with 3% and less are not. In at least four of the studies chosen in this systematic review, the cutoff value for the AHI score was 3%. Kendzierska et al. [75] published a study that suggests OSA-related factors other than AHI were shown as important predictors of cardiovascular outcome - this includes PSG characteristics such as sleep time spent with SaO2 less than 90%, the number of awakenings, mean heart rate and a few others. Other researchers look into OSA phenotypes not captured by traditional stratification by AHI [76].

Only a few studies considered more than one CVC, and in most cases, the data provided was on the prevalence of hypertension. Some studied populations were characterized by a cardiovascular disorder, such as stable ischemic stroke patients or those after myocardial infarction. It is unlikely that all cardiovascular complications are equally connected to oxidative stress and vice versa. The importance of oxidative stress may be different between hypertension and coronary disease or other CVC and should become a premise of future studies.

There was little to no information about subjects’ duration of CVC or the usage of drugs, either for diagnosed cardiovascular disease, hypertension or other comorbidities. A patient that is getting proper treatment may have different results of oxidative stress biomarkers than the one that is not taking any medications, or his treatment is improper or insufficient. Moreover, there could be a confounding effect of CVC drugs on the levels and concentrations of studied biomarkers. To better inspect the relationship between CVC and oxidative stress, studies should provide patients’ full history.

There were some difficulties regarding the extraction of data from the studies. In one article [69], the authors provided biometric characteristics only for all patients combined. Two articles [53,62] presented their oxidative stress biomarkers data only in the form of a graph without providing the numeric results. In one other article [67], CVC was not included separately, but as a part of the group of cardiovascular risk factors.

Lastly, due to the nature of case–control and cohort studies, there is an inability to measure the duration of oxidative stress and its influence on CVC. One time measures of biomarker levels may be insufficient to assess whether the patients are in the continual state of oxidative stress or periodically, e.g., only after the night.

As for future research, longitudinal, prospective studies on OSA patients free of cardiovascular complications that measure the markers of oxidative stress are needed to fully establish the role of oxidative stress in the development of cardiovascular complications.

## 5. Conclusions

The emerging evidence from controlled studies suggests that patients suffering from OSA with cardiovascular complications show the extent of oxidative stress, measured by various biomarkers, similar to OSA patients free of cardiovascular complications.

## Figures and Tables

**Table 1:** Chosen biomarkers of oxidative stress.

Biomarker	Relation to Oxidative Stress
Reactive oxygen species	Reactive oxygen species (ROS) are oxygen molecules with unpaired electrons that interfere with nucleic acids, proteins and lipids structure due to high reactivity and giving rise to cytotoxic tissue damage. ROS are produced by fluctuations in oxygen saturation [14].
Total oxidant status	Total oxidant status (TOS) is a form of presenting the total oxidation state of the sample (plasma, serum, etc.) by conducting a redox reaction. Higher generation of ROS results in higher TOS [15].
Total antioxidant status	Total antioxidant status (TAS) is a form of presenting the total antioxidation state of the sample (plasma, serum, etc.) by conducting a redox reaction. Higher generation of ROS results in lower TAS [16].
Endothelial nitric oxide synthase	Endothelial nitric oxide synthase (eNOS) is the main producer of nitric oxide (NO). eNOS shows a positive correlation with oxidative stress [17,18].
Nitric oxide Metabolites	Nitric oxide metabolites (NOx) are the final metabolites of NO. These metabolites, including nitrites and nitrates, are considered to be involved in tissue injury and exhibit a negative correlation with oxidative stress [19].
Nitric oxide	Nitric oxide (NO) is a form of free radical that plays a key role in the physiological regulation and protection of the cardiovascular system. NO is an antioxidant that reacts with ROS and produces cytotoxic metabolites such as peroxynitrite; therefore, it exhibits a negative correlation with oxidative stress [17,18].
Malondialdehyde	Malondialdehyde (MDA) is a product of the reaction between polyunsaturated fatty acids (PUFAs) and reactive oxygen species (ROS) [20].
Reactive oxygen metabolites	Reactive oxygen metabolites (ROMs) are partially reduced oxygen species produced by oxidases such as xanthine oxidase or NADPH oxidase. They play an important role in mediating cellular injury [21].
Thiobarbituric acid reactive substances	Thiobarbituric acid reactive substances (TBARS) are the products of the reaction between thiobarbituric acid (TBA) and malondialdehyde (MDA), categorized as one of the simplest ways to measure MDA, which is the product of the reaction between PUFAs and ROS [22].
8-hydroxy-2′-deoxyguanosine	8-hydroxy-2′-deoxyguanosine (8-OHdG) is generated during the process of repairing ROS-mediated DNA damages. Higher levels of ROS are related to increased DNA damages, and as a result, more 8-OHdG is generated [23].
F2-isoprostane (8-isoprostane)	F2-isoprostanes (F2-IsoPs) are formed from arachidonic acid (AA) oxidized by free radicals in lipid peroxidation reaction [24].
Superoxide dismutase	Superoxide dismutase (SOD) is an antioxidant enzyme that protects cells from the toxic effects of reactive oxygen species (ROS) [25].
Glutathione	Glutathione (GSH) is involved in a diverse range of processes that include cell growth and programmed death. Serves as an antioxidant by interacting with ROS [26].
Glutathione peroxidase	Glutathione peroxidase (GPx) is a family of enzymes that play an important role in hydrogen peroxide and lipid hydroperoxide detoxification [27].
Serum paraoxonase 1	Paraoxonase-1 (PON-1) is an HDL-bound esterase enzyme that hydrolyzes lipid peroxides, protecting low-density lipoproteins (LDLs) from free radicals [28].
Advanced glycation end products	Advanced glycation end products (AGEs) are lipids or proteins that become glycated and oxidized after repeated contact of free radicals with reducing sugars or aldehydes [29].
Advanced oxidation protein products	Advanced oxidation protein products (AOPPs) are formed from oxidation-modified albumin, which is their main source. They can promote oxidative stress and inflammation [30].
Asymmetric dimethylarginine	Asymmetrical dimethylarginine (ADMA) is an inhibitor of nitric oxide synthase (NOS) and thus inhibits nitric oxide (NO) synthesis [31].
Homocysteine	Homocysteine (Hcy) is an amino acid in which metabolic imbalance can lead to protein, nucleic acid and carbohydrate oxidation [32].
Oxidized low-density lipoprotein	oxidized low-density lipoprotein (oxLDL) is a product of the reaction between ROS and LDL and is considered to play an important role in atherogenesis [33].
Lectin-like oxidized low-density lipoprotein receptor-1	Lectin-like oxidized low-density lipoprotein receptor-1 (LOX-1) in endothelial cells is a crucial receptor responsible for binding and uptake of oxidized low-density lipoprotein (oxLDL) [34].
Total antioxidant capacity	Total antioxidant capacity (TAC) is characterized as the moles of oxidants neutralized by one liter of plasma and serves as a biomarker that measures the body fluids’ antioxidant potential, including synergistic redox interactions [35].
Ferric-reducing antioxidant power	Ferric-reducing antioxidant power (FRAP) is defined as plasma ability to reduce iron complexes and reflects plasma antioxidant potential [36].
Uric acid	Uric acid (UA) is an end product of purine metabolism originating from the oxidation of purine compounds. Uric acid inhibits lipid peroxidation and is considered to be a powerful antioxidant [37].
Non-protein--bound iron	Non-protein-bound iron (NPBI) is a form of low molecular mass iron that interacts with superoxide and hydrogen peroxide, causing the release of hydroxyl radical (OH·), which is an immensely destructive oxidizing species [38].
Catalase	Catalase is a heme protein that decomposes hydrogen superoxide and provides the first line of defense against reactive oxygen metabolites (ROMs) [21].
Matrix metalloproteinases	Matrix metalloproteinases (MMPs) are enzymes that take part in proteolytic remodeling of the extracellular matrix. They interact with ROS, which can disrupt a protein conformation exposing the catalytic site of MMP and increasing MMPs activity [39].

**Table 2:** PICO(s) question.

Do Patients with Obstructive Sleep Apnea Demonstrate an Extent Oxidative Stress Correlated with Cardiovascular Complications?	
Population	Patients with OSA defined as AHI > 5 in polysomnographic sleep study (PSG)
Indicator	Oxidative stress markers/antioxidative markers
Control	Groups of patients without OSA (AHI < 5 in PSG), other OSA patients
Outcome	Cardiovascular complications, such as hypertension. myocardial infarction, stroke, ischemic heart disease, heart failure
Study design	Peer-reviewed English articles Adult (>18 years) human subjects Case–control/observational studies Studies comparing oxidative stress biomarkers between OSA and control groups and providing data on cardiovascular complications

**Table 3:** Selected studies and quality assessment.

Author	Year	Oxidative Stress Biomarkers	AHI Scoring Criteria	OSA Case Definition	CVC Reported	Population	Newcastle–Ottawa Scale *		
							Selection	Comparability	Exposure
**OSA vs non-OSA control**									
Anunciato et al. [53]	2013	Plasma NO	PSG with no oxygen desaturation cutoff value	AHI > 5	HT	Elderly hypertensive	★▢★★	★ ★	★★★
Cherneva et al. [54]	2016	Urinary 8-isoprostane	PSG apnea or hypopnea 3% ↓SpO2 or arousal >10 s	AHI > 5	HT, CAD	ESS < 11	★▢▢★	★★	★★★
de Lima et al. [55]	2010	NOx, superoxides	PSG apnea or hypopnea 4% ↓SpO2	AHI > 20	HT, CAD	Obese (BMI > 30)	★▢▢★	★★	★★★
Del Ben et al. [56]	2012	Urinary 8-isoprostanes, NOx	PSG apnea or hypopnea 4% ↓SpO2 and arousal	AHI > 5	HT	Metabolic disorders + snoring	★▢▢★	★★	★★★
Monneret et al. [57]	2012	TAC, GSH, GPx, Hcy, ur 15-F2t-isoprostane	PSG apnea or hypopnea 4% ↓SpO2 or EEG arousal	AHI > 10	HT, IHD	Non-obese MetS	★▢▢★	★★	★★★
Piérola et al. [58]	2011	Plasma 8-isoprostanes	PSG apnea or hypopnea 4% ↓SpO2 or arousal	AHI > 10	HT	Suspected of having OSA	★★▢★	▢▢	★★★
Wang et al. [59]	2010	Hcy, MDA, GSH	PSG apnea or hypopnea 3% ↓SpO2	AHI > 5	HT	Elderly and non-elderly	★★▢★	★★	★★★
Ortaç Ersoy et al. [60]	2014	NOx, Hcy, TAC	PSG with no oxygen desaturation cutoff value	AHI > 5	CAD	Patients hospitalized after acute MI	★▢▢★	★▢	★★★
**severe OSA vs non-severe OSA**									
Chen et al. [61]	2015	TAC, urinary 8-OHdG	PSG apnea or hypopnea 3% ↓SpO2 or arousal	severe AHI > 30	HT	Stable ischemic stroke patients	★▢▢★	▢▢	★★★
Gille et al. [62]	2017	8-OHdG, MMP-7	PSG with no oxygen desaturation cutoff value	AHI ⩾ 15	HT, IHD, S/TIA	Patients with idiopathic pulmonary fibrosis	★▢▢★	★★	★★★
Yadav et al. [63]	2014	PON1	PSG with no oxygen desaturation cutoff value	mean AHI = 21.3	HT	Obese (BMI > 40)	★▢▢★	★★	★★★
Yamauchi et al. [64]	2005	Urinary 8-OHdG	PSG with no oxygen desaturation cutoff value	severe AHI > 30	HT	Suspected of having OSA	★★▢★	▢★	★★★
Yardim-Akaydin et al. [65]	2013	MDA, 8-OHdG,	PSG apnea or hypopnea 4% ↓SpO2	AHI > 5	HT	Patients with and without MetS	★▢▢★	★★	★★★
**OSA with CVC vs. OSA without CVC**									
Feres et al. [66]	2015	oxLDL	PSG with no oxygen desaturation cutoff value	AHI ⩾ 15	HT	Patients suspected of having OSA	★★★★	▢★	★★★
İn et al. [67]	2015	ADMA	PSG apnea or hypopnea 3% ↓SpO2 or arousal	AHI > 5	HT	OSA patients with and without CV risk factors	★★▢★	▢★	★★★
Lavie et al. [68]	2004	PON1, TBARS, PD	PSG with no oxygen desaturation cutoff value. RDI	RDI > 10 + symptoms	HT, IHD, MI, CVA,	Untreated OSA patients	★★▢★	▢★	★★★
Murri et al. [69]	2009	CAT, GPx, SOD, TAC	PSG apnea or hypopnea 4% ↓SpO2	AHI > 10 and ESS > 10	HT	OSA patients that required CPAP	★▢▢★	▢▢	★★★

AHI, apnea/hypopnea index; CVC, cardiovascular complication; PSG, polysomnography; HT, hypertension, CAD, coronary artery disease; NO, nitrogen oxide; SpO2, blood oxygen saturation level; ESS, Epworth sleepiness scale; NOx, nitrate/nitrate; TAC, total antioxidant capacity; GSH, glutathione; GPx, glutathione peroxidase; Hcy, homocysteine; EEG, electroencephalography; IHD, ischemic heart disease; MetS, metabolic syndrome; ADMA, asymmetric dimethylarginine; MDA, malondialdehyde; 8-OHdG, 8-oxo-2′-deoxyguanosine; MMP-7, matrix metalloproteinase-7; S, stroke; TIA, transient ischemic attack; CHF, congestive heart failure; PVD peripheral vascular disease; PON1, paraoxonase 1; oxLDL, oxidated low-density lipoprotein; CV, cardiovascular; TBARS, thiobarbituric acid reactive substances, PD, peroxides; CAT, catalase; CPAP, continuous positive airway pressure, SOD, superoxide dismutase; MI, myocardial infarction.* Stars and squares represent Newcastle-Ottawa Scale (NOS) Score in three categories in the following order: Selection contains four criteria: Is the case definition adequate (OSA)?; representativeness of the cases; selection of controls (volunteers, not referred for a sleep study); definition of controls. Comparability scores: controls and cases are matched by age and BMI; controls and cases are matched by the prevalence of CVD/AHI for non-CVD OSA controls. Exposure contains three criteria: ascertainment of exposure (oxidative stress); same method of ascertainment for cases and controls: nonresponse rate. For every point awarded in NOS, a star is given, otherwise it is shown as a square.

**Table 4:** Oxidative stress characteristics in obstructive sleep apnea (OSA) group vs. non-OSA group.

Author	Study and Control Characteristics							Biomarker Levels	
	Sample Size (*n*)	Age ± SD	Male (%)	Mean BMI ± SD	Mean AHI ± SD	CVC (%)	Differences in Groups *	Results	Differences in Groups ^
Anunciato et al. [53]	**Study Group—Hypertensive Patients wWith OSA**							No	NO Data presented graphically; therefore, parametric results cannot be displayed on this table.	No
	25	67.0 ± 6.5	-	30.3 ± 4.8	29.0 ± 13.7		HT: 100			
	**Control group—Hypertensive Patients Without OSA**									
	12	67.8 ±6.8	-	29.0 ± 5,0	3.1 ± 1.6	HT: 100				
Cherneva et al. [54]	**Study Group—Patients with Minimally Symptomatic OSA**							No	8-isoprostane: 0.091 ± 0.007 pg/mkmol creatinine	8-isoprostane
	86	54.66 ±11.59	88.4	31.1±4.63	38.94 ± 13.53	HT: 41.21				
	**Control Group—Patients Without OSA**								8-isoprostane: 0.078±0.004 pg/mkmol creatinine	
	45	52.17 ± 8.95	80	29.8 ± 3.92	4.10 ± 1.90	HT: 44.53				
De Lima et al. [55]	**Study Group—Patients with OSA Not Treated With CPAP**							No	NOx: 30.3 ± 7.9 µM	NOx
	10	57 ± 10.5	100	34.1 ± 1.3	29.5 ± 3.7	HT: 17.2				
	**Control Group—Patients Without OSA**								NOx: 50.5 ± 2.9 µM	
	10	56.8 ± 4.7	100	33.1 ± 2.5	3.6 ± 0.1	13.8				
Del Ben et al. [56]	**Study Group—Severe OSA**							BMI	8-isoprostane: 337.6 ± 74.5 pg/mg creatinine NOx: 23.6 ± 16.0 uM/mL	8-isoprostane (severe OSA vs control and mild/moderate OSA)
	30	56.6 ± 9.8	83.3	32.8 ± 5.1	42.8 ± 14.2	HT: 66.7				
	**Study Group—Mild/Moderate OSA**								8-isoprostane: 289.5 ± 77.0 pg/mg creatinine NOx: 27.0 ± 12.5 uM/mL	
	61	53.2 ± 11.7	70.5	30.5 ± 4.5	14.6 ± 8.0	HT: 60.7				
	**Control Group—Snorers**								8-isoprostane: 284.0 ± 77.3 pg/mg creatinine NOx: 27.1 ± 14.6 uM/mL	
	47	51.3 ± 11.7	66.0	29.3 ± 3.9	1.2 ± 1.4	HT: 51.1				
Monneret et al. [57]	**Study Group—OSA + Metabolic Syndrome Patients**							No	TAS: 1.59 ± 0.21 mmol/L GPx: 51.0 ± 9.7 U/g Hb GSH: 1022 ± 178 µmol/L Hcy: 12.8 ± 3.8 µmol/L F2-isoprostane: 21.2 ± 10.4 ng/mmol creatinine	Hcy
	26	61.5 ± 5.0	65.4	29.8 ± 3.7	31.7 ± 20.5	HT: 88 IHD: 8				
	**Control Group—Metabolic Syndrome Patients**								TAS: 1.59 ± 0.10 mmol/L GPx: 51.4 ± 11.4 U/g Hb GSH: 1040 ± 137 µmol/L Hcy: 9.5 ± 2.5 µmol/L F2-isoprostane: 16.5 ± 4.5 ng/mmol creatinine	
	9	59.7 ± 3.4	33.3	29.1 ± 2.6	5.4 ± 3.6	HT: 89 IHD: 0				
Pierola et al. [58]	**Study Group**							Age, BMI, CVC	8-isoprostane: 12.10 (IQR: 6.23–23.98) ng/dL	8-isoprostane
	427	51 ± 12	82	31.4 ± 6	48 ± 24	HT: 49				
	**Control Group**								8-isoprostane: 5.07 (IQR: 1.41–11.56) ng/dL	
	139	45 ± 12	74	27.9 ± 5	3 ± 2	HT: 24				
Wang et al. [59]	**Study Group—Elderly With OSA**							No	Hcy: 18.70 ± 4.73 µmol/L MDA: 6.18 ± 1.23 nmol/mL GSH: 8.79 ± 2.68 mg/L	Hcy, MDA, GSH
	32	65.8 ± 7.2	90	23.34 ± 2.36	38.67 ± 21.28	HT: 15.63				
	**Control Group—The Elderly Without OSA**								Hcy: 11.13 ± 3.05 µmol/L MDA: 5.04 ± 0.69 nmol/mL GSH: 6.68 ± 3.13 mg/L	
	29	69.4 ± 4.2	93.1	26.85 ± 2.9	2.93 ± 1.09	HT: 13.79				
	**Study Group—Non-Elderly with OSA**								Hcy: 10.84 ± 2.56 µmol/L MDA: 5.18 ± 1.51 nmol/mL GSH: 6.42 ± 2.00 mg/L	Hcy, MDA, GSH
	51	42.7 ± 8.3	90.2	28.36 ± 3.51	45.51 ± 25.20	HT: 13.73				
	**Control Group—Non-Elderly without OSA**								Hcy: 8.90 ± 1.23 µmol/L MDA: 4.12 ± 1.09 nmol/mL GSH: 4.18 ± 1.19 mg/L	
	23	44.7 ± 12.3	87	25.13 ± 3.61	3.42 ± 2.10	HT: 13.04				
Ortaç Ersoy et al. [60]	**OSA**							AHI (OSA group vs OSA + CAD group)	NOx: 66.7 ± 20.3 μmol/L Hcy: 14.3 ± 2.7 μmol/L TAC: 2.2 ± 0.4 μmol/L	No
	15	53.7 ± 8.1	-	27.5 ± 3.2	41.6 ± 27.4	CAD: 0				
	**CAD**								NOx: 77.6 ± 16.6 μmol/L Hcy: 16.4 ± 6.8 μmol/L TAC: 2.2 ± 0.2 μmol/L	
	15	53.2 ± 7.2	-	25.7 ± 3.6	1.9 ± 1.2	CAD: 100				
	**OSA + CAD**								NOx: 83.7 ± 26.9 μmol/L Hcy: 16.9 ± 4.6 μmol/L TAC: 2.2 ± 0.3 μmol/L	
	12	54.7 ± 6.9	-	28.0 ± 3.4	16.2 ± 9.7	CAD: 100				
	**Normal**								NOx: 73.8 ± 17.8 μmol/L Hcy: 12.3 ± 2.5 μmol/L TAC: 2.1 ± 0.3 μmol/L	
	10	48.8 ± 6.6	-	26.2 ± 1.6	2.7 ± 1.9	CAD: 0				

* *p* < 0.05 when comparing age. BMI.% CVC or biomarker level; ^ *p* < 0.05 when comparing biomarkers; IQR–interquartile range; SD, standard deviation; BMI, body mass index; AHI, apnea/hypopnea index; CVC, cardiovascular complication; HT, hypertension,; NO, nitrogen oxide; CPAP, continuous positive airway pressure; NOx, nitrate/nitrate; GSH, glutathione; GPx, glutathione peroxidase; TAS, total antioxidant status; Hcy, homocysteine; IHD, ischemic heart disease; MDA, malondialdehyde; TAC, total antioxidant capacity; CAD, coronary artery disease.

**Table 5:** Oxidative stress characteristics in severe OSA group vs. non-severe OSA group.

Author	Study and Control Characteristics								Biomarker Levels	
	Sample Size (*n*)	Age ± SD	Male (%)	Mean BMI ± SD	Mean AHI ± SD		CVC (%)	Differences in Groups *	Results	Differences in Groups ^
**Chen et al.** [61]	**AHI < 30**							Age, HT	TAC: 596.3 ± 118.3 μmol/L 8-OHdG: 33.7 (IQR: 27.2–49.7) ng/mgcr	No
	34	58.1 ± 13.9	61.8	24.9 ± 4.2	18.1 (10.3–24.5)	Stroke: 100HT: 74				
	**AHI > 30**								TAC: 606.1 ± 102.6 μmol/L 8-OHdG: 39.5 (IQR: 29.6–51.5) ng/mgcr	
	58	66.5 ± 11.0	72.4	24.9 ± 3.7	50.9 (38.3–60.9)	Stroke: 100 HT: 91				
**Gille et al.** [62]	**No OSA**							Age (No OSA vs moderate OSA)	8-OHdG MMP-7 Data presented graphically; parametric results cannot be displayed in this table.	8-OHdG (moderate and severe OSA vs. mild and No OSA), MMP-7 (severe OSA vs. moderate, mild and No OSA)
	5	61 ± 10.9	60	27.7 ± 4.3	3 ± 1.7	41.2 (HT: 35.3, IHD: 11.8, Stroke/TIA: 0) ^$^				
	**Mild OSA**									
	12	67.6 ± 7.5	75	27.8 ± 2.5	10.8 ± 2.6	41.2 (HT: 35.3, IHD: 11.8, Stroke/TIA: 0) ^$^				
	**Moderate OSA**									
	10	72.1 ± 7.7	90	27.3 ± 4	22.6 ± 3.4	40 (HT: 30, IHD: 30, Stroke/TIA: 0)				
	**Severe OSA**									
	18	69.8 ± 8.6	94	28.6 ± 3.7	60.5 ± 25	100 (HT: 55.6, IHD: 61.1, Stroke/TIA: 16.7)				
**Yadav et al.** [63]	**High AHI**							No	PON1:101 ± 64 nmol/mL/min	PON1
	20	49 ± 10	15	52 ± 6	21.3 (13.5–45.7)	HT: 65				
	**Low AHI**								PON1:186 ± 68 nmol/mL/min	
	21	45 ± 9	20	50 ± 8	4.3 (3.7–6.8)	HT: 50				
**Yamauchi et al.** [64]	**Non-Severe OSA**							BMI	8-OHdG: 8.5 ± 2.4 ng/mL/creatinine mg/mL	8-OHdG
	70	49.3 ± 12.2	82.9	25.9 ± 3.8	12.1 ± 9.3	HT: 21.4				
	**Severe OSA**								8-OHdG: 9.5 ± 2.5 ng/mL/creatinine mg/mL	
	58	48.8 ± 11.0	98.0	29.5 ± 5.3	60.7 ± 19.2	HT: 34.5				
**Yardim-Akaydin et al.** [65]	**Controls**							Age (moderate and severe apnea vs. mild and control), BMI (severe apnea vs. moderate, severe apnea and control), HT (severe apnea vs. control)	MDA: 2.24 ± 0.91 µM 8-OHdG: 25.63 ± 8.14 nM/creatinine mM	MDA (controls vs. mild, moderate and severe apnea), 8-OHdG (mild apnea vs. severe apnea; controls vs. mild and moderate apnea)
	25	43.4 ± 8.2	56	27.2 ± 3.2	2.7 ± 1.2	HT: 0				
	**Mild Apnea**								MDA: 3.01 ± 1.18 µM 8-OHdG: 38.20 ± 19.03 nM/creatinine mM	
	28	43.1 ± 9.7	64.29	30.03 ± 4.8	9.5 ± 3.3	HT: 14.3				
	**Moderate Apnea**								MDA: 3.00 ± 0.89 µM 8-OHdG: 32.08 ± 12.69 nM/creatinine mM	
	30	52.9 ± 11.8	67	30.0 ± 4.0	22.5 ± 4.3	HT: 13.3				
	**Severe Apnea**								MDA: 3.33 ± 1.11 µM 8-OHdG: 29.40 ± 16.35 nM/creatinine mM	
	59	51.2 ± 10.7	72.88	33.0 ± 5.8	56.3 ± 22.3	HT: 32.2				

* *p* > 0.05 when comparing age, BMI, CVC/AHI in OSA controls; ^ *p* < 0.05 when comparing biomarkers; author provided data for both No OSA and mild OSA groups combined. IQR—interquartile range; SD, standard deviation; BMI, body mass index; AHI, apnea/hypopnea index; CVC, cardiovascular complication; HT, hypertension; TAC, total antioxidant capacity; 8-OHdG, 8-oxo-2′-deoxyguanosine; IQR, interquartile range; TIA, transient ischemic attack; IHD, ischemic heart disease; MMP-7, matrix metalloproteinase-7; PON1, paraoxonase 1; MDA, malondialdehyde. ^$^ Author provided biometrical data of the whole studied population, not providing data after dividing patients into hypertensive and normotensive groups.

**Table 6:** Oxidative stress characteristics in the OSA group with cardiovascular complications (CVC) vs OSA without CVC group.

Author	Study and Control Characteristics									Biomarker Levels	
	Sample Size (*n*)	Age ± SD	Male (%)	Mean BMI ± SD		Mean AHI ± SD		CVC (%)	Differences in Groups *	Results	Differences in Groups ^
Feres et al. [66]	**OSA + comorbidities**								Age (OSA + comorbidities and OSA vs control), BMI (OSA + comorbidities vs control)	oxLDL: 89.42 ± 29.53 U/L	No
	48	53.77 ± 8.03	36	28.97 ± 3.11		25.02 ± 15.97		HT: 41.6			
	**OSA**									oxLDL: 83.63 ± 25.2 U/L	
	24	50.75 ± 7.43	50	28.13 ± 3.74		23.07 ± 18.59		HT: 0			
	**Control**									oxLDL: 69.46 ± 24.79 U/L	
	27	44.38 ± 7.03	38	25.79 ± 4.49		1.98 ± 1.69		HT: 0			
İn et al. [67]	**OSA with CV Risk Factors (obesity, hypercholesterolemia, DM, HT and smoking)**								Age (OSA with CV risk factors vs OSA without CV risk factors and control subjects), BMI (OSA with CV risk factors vs OSA without CV risk factors and control subjects)	ADMA: 0.64 ± 0.12 µmol/L	ADMA (control subjects vs OSA with and without CV risk factors)
	26	57.5 ± 10.8	65.4	38.5 ± 8.6		34.8 ± 23.3		80.7^#^			
	**OSA without CV Risk Factors**									ADMA: 0.67 ± 0.13 µmol/L	
	14	45.9 ± 8.8	85.7	28.5 ± 1.6		28.3 ± 24.6		0^#^			
	**Control Subjects**									ADMA: 0.47 ± 0.19 µmol/L	
	20	49.3 ± 10.9	70	26.7 ± 2.3		1.9 ± 1.2		0			
Lavie et al. [68]	**OSA + CVD**								Age (OSA + CVD vs OSA − CVD and controls), BMI (OSA + CVD vs OSA − CVD and controls; OSA − CVD vs controls)	TBARS: 18.6 ± 7.3 nmol MDA/mL PON1:79.5 ± 13.6 U/min/mL	TBARS (controls vs OSA + CVD and OSA − CVD), PON1 (controls vs OSA + CVD)
	59	58.5 ± 11.3	83.0	30.6 ± 5.5		31.3 ± 18.5		IHD: 37.9 MI: 8.5 HT: 75.9			
	**OSA - CVD**									TBARS: 17.2 ± 6.3 nmol MDA/mL PON1:86.7 ± 17.6 U/min/mL	
	55	46.8 ± 10.2	85.5	28.4 ± 3.5		26.9 ± 13.8		IHD: 0 MI: 0 HT: 0			
	**Controls**									TBARS: 12.9 ± 3.5 nmol MDA/mL PON1:92.1 ± 14.4 U/min/mL	
	30	42.9 ± 13.8	90.0	26.0 ± 3.2		8.2 ± 2.6		IHD: 3.3 MI: 0 HT: 13.3			
Murri et al. [69]	**Hypertensive OSA**								Data provided for the whole studied population only ^$^	CAT: 2.52 ± 1.15 nmol/min/m GPx: 20.49 ± 5.52 mmol/min/mL SOD: 0.066 ± 0.025 U/mL TAC: 3.605 ± 1.338 mmol/L	CAT, TAC
	60	55.32 ± 11.28 ^$^	-	32.21 ± 5.19 ^$^	54.77 ± 19.34 ^$^		HT: 100				
	**Normotensive OSA**									CAT: 2.66 ± 1.19 nmol/min/m GPx: 23.34 ± 7.64 mmol/min/mL SOD: 0.072 ± 0.021 U/mL TAC: 4.127 ± 1.256 mmol/L	
	18	55.32 ± 11.28 ^$^	-	32.21 ± 5.19 ^$^	54.77 ± 19.34 ^$^		HT: 0				
Ortaç Ersoy et al. [60]	**OSA**								AHI (OSA group vs OSA + CAD group)	NOx: 66.7 ± 20.3 μmol/L Hcy: 14.3 ± 2.7 μmol/L TAC: 2.2 ± 0.4 μmol/L	No
	15	53.7 ± 8.1	-	27.5 ± 3.2	41.6 ± 27.4		CAD: 0				
	**CAD**									NOx: 77.6 ± 16.6 μmol/L Hcy: 16.4 ± 6.8 μmol/L TAC: 2.2 ± 0.2 μmol/L	
	15	53.2 ± 7.2	-	25.7 ± 3.6	1.9 ± 1.2		CAD: 100				
	**OSA + CAD**									NOx: 83.7 ± 26.9 μmol/L Hcy: 16.9 ± 4.6 μmol/L TAC: 2.2 ± 0.3 μmol/L	
	12	54.7 ± 6.9	-	28.0 ± 3.4	16.2 ± 9.7		CAD: 100				
	**Normal**									NOx: 73.8 ± 17.8 μmol/L Hcy: 12.3 ± 2.5 μmol/L TAC: 2.1 ± 0.3 μmol/L	
	10	48.8 ± 6.6	-	26.2 ± 1.6	2.7 ± 1.9		CAD: 0				

* *p* < 0.05 when comparing age, BMI, CVC/AHI in OSA controls; ^ *p* < 0.05 when comparing biomarkers; ^#^ cardiovascular risk factors in the reported group is shown as a percentage of patients presenting at least one of the following: obesity, hypercholesterolemia, diabetes mellitus, hypertension and smoking. 7.7% of the patients had hypertension without other risk factors. 73.1% had more than one risk factor. Unable to extract exact data regarding the prevalence of CVC; ^$^ Author provided biometrical data of the whole studied population, not providing data after dividing patients into hypertensive and normotensive groups. SD, standard deviation; BMI, body mass index; AHI, apnea/hypopnea index; CVC, cardiovascular complication; HT, hypertension; oxLDL, oxidized low-density lipoprotein; CV, cardiovascular; DM, diabetes mellitus; ADMA, asymmetric dimethylarginine; CVD, cardiovascular disease; IHD, ischemic heart disease; MI, myocardial infarction; TBARS, thiobarbituric acid reactive substances; MDA, malondialdehyde; PON1, Serum paraoxonase 1; CAT, catalase; TAC, total antioxidant capacity; GPx, glutathione peroxidase; SOD, Superoxide dismutase; CAD, coronary artery disease; NOx, nitrate/nitrate; Hcy, homocysteine.
